# Adropin Stimulates Proliferation and Inhibits Adrenocortical Steroidogenesis in the Human Adrenal Carcinoma (HAC15) Cell Line

**DOI:** 10.3389/fendo.2020.561370

**Published:** 2020-10-08

**Authors:** Ewelina Stelcer, Paulina Milecka, Hanna Komarowska, Karol Jopek, Marianna Tyczewska, Marta Szyszka, Marta Lesniczak, Wiktoria Suchorska, Karlygash Bekova, Beata Szczepaniak, Marek Ruchala, Marek Karczewski, Tomasz Wierzbicki, Witold Szaflarski, Ludwik K. Malendowicz, Marcin Rucinski

**Affiliations:** ^1^Department of Histology and Embryology, Poznan University of Medical Sciences, Poznan, Poland; ^2^Radiobiology Lab, Greater Poland Cancer Centre, Poznan, Poland; ^3^Department of Electroradiology, Poznan University of Medical Sciences, Poznan, Poland; ^4^Department of Endocrinology, Metabolism and Internal Medicine, Poznan University of Medical Sciences, Poznan, Poland; ^5^West Kazakhstan Marat Ospanov Medical University, Aktobe, Kazakhstan; ^6^Department of General and Transplantation Surgery, Poznan University of Medical Sciences, Poznan, Poland; ^7^Department of General, Endocrinological and Gastroenterological Surgery, Poznan University of Medical Sciences, Poznan, Poland

**Keywords:** adrenal, adropin, TGF-beta, HAC15, GPR19

## Abstract

Adropin is a multifunctional peptide hormone encoded by the *ENHO* (energy homeostasis associated) gene. It plays a role in mechanisms related to increased adiposity, insulin resistance, as well as glucose, and lipid metabolism. The low adropin levels are strongly associated with obesity independent insulin resistance. On the other hand, overexpression or exogenous administration of adropin improves glucose homeostasis. The multidirectional, adropin-related effects associated with the regulation of metabolism in humans also appear to be attributable to the effects of this peptide on the activity of various elements of the endocrine system including adrenal cortex. Therefore, the main purpose of the present study was to investigate the effect of adropin on proliferation and secretory activity in the human HAC15 adrenal carcinoma cell line. In this study, we obtained several highly interesting findings. First, GPR19, the main candidate sensitizer of adrenocortical cells to adropin, was expressed in HAC15 cells. Moreover, GPR19 expression was relatively stable and not regulated by ACTH, forskolin, or adropin itself. Our findings also suggest that adropin has the capacity to decrease expression levels of steroidogenic genes such as steroidogenic acute regulatory protein (*StAR*) and *CYP11A1*, which then led to a statistically significant inhibition in cortisol and aldosterone biosynthesis and secretion. Based on whole transcriptome study and research involving transforming growth factor (TGF)-β type I receptor kinase inhibitor we demonstrated that attenuation of steroidogenesis caused by adropin is mediated by the TGF-β signaling pathway likely to act through transactivation mechanism. We found that HAC15 cells treated with adropin presented significantly higher proliferation levels than untreated cells. Using specific intracellular inhibitors, we showed that adropin stimulate proliferation via ERK1/2 and AKT dependent signaling pathways. We have also demonstrated that expression of GPR19 is elevated in adrenocortical carcinoma in relation to normal adrenal glands. High level of GPR19 expression in adrenocortical carcinoma may constitute a negative prognostic factor of disease progression.

## Introduction

Adropin is a peptide hormone encoded by the ENHO (energy homeostasis associated) gene, which is generally expressed in the liver, pancreas, and central nervous system (1). The encoded peptide is composed of 76 amino acids and residues 1-33 (cleaved during secretion) encode a secretory signal peptide sequence (2). This amino acid sequence is characterized by very high interspecies homology: it is identical in mice, rats and humans (3). ENHO expression in the brain is observed in ventromedial and lateral hypothalamic nuclei regulating appetite and autonomic functions. In turn, lower ENHO expression is characteristic in the liver, lung, kidney, ileum, and some endocrine glands. Hepatic ENHO expression is correlated with the expression of genes involved in glucose and lipid metabolism (4, 5). Furthermore, human studies showed that adropin may contribute to cholesterol homeostasis. It was found that in men, plasma adropin concentration is inversely correlated with low-density lipoprotein (LDL-C) cholesterol (6). Physiological serum adropin levels are still poorly defined, however, the adropin concentration in blood depends on a well-defined factors. Low adropin levels are strongly associated with pathological conditions such as insulin resistance, which is independent of the presence of obesity, dyslipidemia, hepatic steatosis, or increased fat mass (7). Overexpression or exogenous administration of adropin improves glucose homeostasis and increases capillary density and blood flow in mouse hind limb ischemia models (8). Moreover, adropin has been reported to have an endothelial protective role through increased eNOS (endothelial nitric oxide synthase) expression. Therefore, it can be assumed that the major function of adropin is to maintain energy homeostasis and insulin response (9). Treatments involving either transgenic overexpression of adropin or systemic adropin treatment clearly attenuate insulin resistance and glucose intolerance in dieting obese mice. In parallel with these observations, adropin knockout mice have been shown to present increased hepatic steatosis, adiposity and insulin resistance (10). By contrast, adropin deficiency correlates with the severity of obesity, dyslipidemia, and insulin resistance (3).

The secretion of adropin into the circulation and its biological effects are still poorly understood. Adropin is an endogenous ligand for GPR19 and adropin-mediated activation of GPR19 stimulates the intracellular MAPK/ERK1/2 signaling pathway, which is essential for the upregulation of E-cadherin and accompanying phenotypic changes in human breast cancer cells (11).

The multidirectional, adropin-related effects associated with the regulation of metabolism in humans also appear to be attributable to the effects of this peptide on the activity of various elements of the endocrine system. The adrenal cortex appears to be among the organs most affected by adropin.

According to the published data, hormonal regulation of adrenal function occurs primarily through activation of G protein-coupled receptors (GPCRs). GPCRs play superior role to many endocrine and neurotransmitter pathways, and their dysfunction contributes to some of the most prevalent human diseases (12). We presume that adropin–next to adiponectin, orexin, leptin, or neuromedin U–constitutes a peptide that affects adrenocortical activity. Given this context, the main purpose of the present study was to investigate the effect of adropin on basic (proliferation) and highly specific (steroidogenesis) biological activity in the HAC15 adrenal carcinoma cell line. The intracellular molecular mechanism of adropin-GPR19 interaction was determined using specific inhibitors and global gene expression study. Finally, the clinical significance of the obtained results was verified by analysis of *ENHO* and *GPR19* gene expression in human normal adrenals in relation to adrenocortical carcinoma.

## Materials and Methods

### Adrenocortical Carcinoma Cell Lines and Treatment

The commercially available HAC15 cell line (ATCC® CRL-3301^TM^, VA, USA) was cultured in a defined medium consisting of DMEM/F-12 without phenol red (Thermo Fisher Scientific, Waltham, MA, USA), 10% Cosmic Calf Serum (Hyclone, GE Healthcare, MA USA), 1% ITS + Premix (Corning, NY, USA), and 1% P/S (Merck Millipore, Darmstadt, Germany). The cells at low passages (p0-p1) underwent starvation in the DMEM/F-12 medium supplemented with 10% charcoal-stripped fetal bovine serum (FBS) (Thermo Fisher Scientific, MA, USA) and 1% P/S for 24 h. Subsequently, the cells were treated with the following compounds: ACTH (Synacthen) 10^−7^ M (Basel, Switzerland), angII 10^−7^ M (Sigma-Aldrich, MO, USA), forskolin 25 μM (Merck Millipore, Germany), and adropin 10^−8^ M (Bachem, Switzerland) for another 24 h. The cells cultured in the medium with 10% charcoal-stripped FBS served as controls. Immediately after 24 h of incubation, the cells and culture media were collected and stored at −80°C for further analysis. The commercially available Y1 cell line (ATCC® CCl-79^TM^, VA, USA) was cultured in defined medium consisting of ATCC-formulated F-12K medium (ATCC® 30-2004^TM^, VA, USA), 2.5% Fetal Bovine Serum (Hyclone, GE Healthcare, MA USA), 15% Donor Horse Serum (Biowest, MO, USA), 1% ITS + Premix (Corning, NY, USA) and 1% P/S (Merck Millipore, Germany).

### Primary Human Adrenal Carcinoma (PAC) Cell Culture

Fresh adrenocortical carcinoma tumor samples obtained during surgery treatment, were cut into several small pieces. Adrenal fragments were further dissociated enzymatically in 25 ml of DMEM F12 without phenol red (Thermo Fisher Scientific, MA, USA) medium containing 0.1% type I collagenase (17018029; ThermoFisher Scientific, MA, USA) for 45 min in a 37°C water bath with intermittent mixing. After digestion, the mixture was filtered through a 70 μm sieve. After that tissue was centrifuged at 4°C, 1,200 rpm for 7 min. The cell pellet was resuspended in DMEM F12 medium with an addition of antibiotic-antimycotic solution and 10% FBS (Hyclone, GE Healthcare, MA USA). Primary cell culture were cultivated at 37°C and 5% CO_2_ in humidified atmosphere up to 70% confluence. The use of postoperative adrenocortical carcinoma tumor samples for the cell culture has been approved by the Bioethics Committee of PUMS (decision no. 255/15).

### Hormone Level Detection

Aldosterone and cortisol concentrations in cell culture media were measured by the ELISA method following the manufacturer's technical protocols (Aldosterone ELISA, cat. no. DE5298 Demeditec Diagnostics GmbH, Kiel, Germany; Cortisol ELISA, cat. no. DEH3388; Corticosterone cat. no. DEV9922 Demeditec Diagnostics GmbH, Kiel, Germany). Absorbance (OD) of each plate well was determined using the Biotek—Synergy 2 microtiter plate reader at 450 nm. Quantitative analysis was carried out using a four-parameter logistic curve (4PL) from “drc” Bioconductor package (13).

### Immunofluorescence

The cells were washed tree times with phosphate buffered saline (PBS) solution (Sigma-Aldrich, MO, USA) and fixed for 20–25 min in 100% methanol. Then, they were rinsed with PBS supplemented with 1% bovine serum albumin (BSA) (Sigma-Aldrich, MO, USA). Then the cells were incubated in PBS containing 1% BSA and 0.2% Triton X-100 (Sigma-Aldrich, MO, USA) for 30 min. After this time, the cells were washed tree times with PBS + 1% BSA. The cells were incubated overnight at 4°C with the following primary antibodies: GPR-19 (ab123014, Abcam, UK) (1:200); CYP11B2 (ab167413, Abcam, UK) (1:250); StAR (8449S, Cell Signaling Technology, MA, USA) (1:250); and CYP11A1 (14217S, Cell Signaling Technology, MA, USA) (1:800). All of primary antibodies were diluted in PBS containing 1% BSA and 0.2% Triton X-100. After incubation with the primary antibodies, the cells were rinsed with PBS + 1% BSA. A secondary antibody–rabbit polyclonal antibody (1:500) (711-546-152) (Jackson ImmunoResearch, PA, USA)–was diluted with PBS+ 1% BSA and incubated for 1 h at 37°C in the dark. Then the cells were washed with PBS+ 1% BSA and stained for 5 min with diamidino-2-phenylindole dye (DAPI) solution (Sigma-Aldrich, MO, USA) in water (1:10,000). Before undergoing microscope analysis, the cells were washed three times with PBS. The acquisition was done using confocal laser scanning microscopy (Olympus FV10i with dedicated 60x water-immersion objective). Z-stack images were collected using Olympus FV10-ASW software. The same laser power (40% for Alexa Fluor® 488 and 12% for DAPI) was used for all images. Densitometric analysis was performed using ImageJ software (ImageJ 1.5q, Wayne Rasband, National Institutes of Health, USA) according to The Open Lab Book protocol (https://theolb.readthedocs.io/en/latest/imaging/measuring-cell-fluorescence-using-imagej.html). The images were measured by fluorescence using the green channel color range. Three images from each group were evaluated. The software was used to calculate integrated density from ten randomly selected areas of 40/40 pixels. Background fluorescence was determined for each image. The corrected total cell fluorescence (CTCF) values were calculated according to the following formula: CTCF = integrated density–(selected area X mean background fluorescence). All CTCF sets (N = 30/group) are shown on the graph and statistically analyzed (Mann–Whitney *U*-test).

### Western Blot Analysis

Total proteins for the Western blot analysis were extracted from the HAC15 cells 24 h after treatment. Cells were collected, washed with PBS, and homogenized with RIPA buffer (ThermoFisher Scientific, MA, USA). After centrifugation at 13,000 rpm at 4°C for 30 min, the supernatant was transferred into new tubes. The concentration of the protein sample was measured using the Pierce BCA Protein Assay Kit (23225, MA, USA). Fifteen micrograms of total protein of each cell extract was resolved by Tris/Glycine/Sodium dodecyl sulfate polyacrylamide gel electrophoresis and transferred to a polyvinylidinedifluoride membrane (1704156, Bio-Rad, CA, USA). Non-specific binding was blocked by incubation in 5% non-fat milk in Tris-buffered saline and Tween 20 at room temperature for 1 h. Blots were then probed overnight at 4°C with anti-GPR 19 1 μg/ml (ab 123014 abcam, UK) and anti-GPADH 1:1000 (5174S, Cell Signaling Technology, MA, USA). Immunoreactive bands were then probed for 1 h at room temperature with the appropriate horseradish peroxidase (HRP)-conjugated secondary anti-Rabbit IgG-HRP 1:2000 (7074S, Cell Signaling Technology, MA, USA). Protein bands were detected by WesternBright Quantum HRP substrate (Advansta, CA, USA) and imaged using a ChemiDoc Imaging Systems (Biorad, CA, USA).

### RNA Extraction

Total RNA isolation was performed according to the modified Chomczynski method with TRI Reagent (Sigma-Aldrich, MO, USA) and the RNeasy Mini Elute Cleanup Kit (Qiagen, Hilden, Germany) in accordance with the manufacturer's instructions. The concentration of isolated RNA was determined by spectrophotometric measurement of absorbance at 260 nm. The purity of the extracted RNA was calculated using an absorbance ratio of 260/280 nm (NanoDrop spectrophotometer, Thermo Scientific, MA, USA). The integrity and quality of the isolated RNA was examined with the Agilent 2100 Bioanalyzer (Agilent Technologies, Inc., Santa Clara, CA, USA). The received RNA integrity numbers (RINs) varied from 8.5 to 10, with an average of 9.2. RNA samples were diluted to 50 ng/μl. One-hundred ng of RNA was dedicated to the microarray study. The remaining total RNA was utilized for the validation of microarray results by qPCR.

### Reverse Transcriptase-PCR and qPCR Analysis

The reverse transcriptase (RT) reaction was carried out using the iScript™ cDNA synthesis kit (Bio-Rad, CA, USA) in a total volume of 20 ul. One μg of total RNA was incubated with Oligo(dT) as primer for 40 min, at a temperature of 45°C. Real-time PCR reactions were carried out using the LightCycler® 480 Probes Master (Roche, Switzerland) and pair of specific primers and probes designed with the Universal Probe Library software (Roche Diagnostics, Switzerland). RT-qPCR reactions were carried out under the following thermal conditions: 10-min denaturation step for the Taq DNA polymerase activation, then by a two-step amplification program repeated 40 times: denaturation at 95°C for 30 s followed by annealing and extension at 64°C for 60 s. The cycle threshold values obtained for the genes of interest were normalized to hypoxanthine-guanine phosphoribosyltransferase gene (HPRT), which previous studies have shown to be one of the most stable reference genes in the adrenal gland (14). The expression level for each examined gene was determined using the formula −2^ΔΔct^. The reaction was carried out in triplicate for each of analyzed genes.

### Microarray Expression Study

The microarray study was performed as described in detail elsewhere (15–18). The total RNA isolated previously was pooled into four samples per group (control, ACTH, forskolin and adropin) treated as described above (see *Cell culture and Treatment* section). The protocol including *in vitro* transcription, biotin labeling, and cDNA fragmentation was performed using the Affymetrix GeneChip IVT Express Kit (Affymetrix, Santa Clara, CA, USA). Then the biotin labeled cDNA were hybridized with the Affymetrix Gene Chip Human Genome U219 microarrays together with appropriate internal controls. The hybridization was performed in the AccuBlockTM Digital Dry Bath hybridization oven (Labnet International, Inc., Edison, NJ, USA) at 45°C for 16 h. Subsequently, the microarrays were washed and stained by means of the Affymetrix GeneAtlas Fluidics Station (Affymetrix, Santa Clara, CA, USA). The microarrays were scanned using the Imaging Station of the GeneAtlas System. Initial analysis of the scanned microarrays was carried out with Affymetrix GeneAtlas TM Operating Software.

### Analysis of Microarray Data

For all bioinformatic analyses we used R statistical programming language supplemented with relevant Bioconductor libraries. Background correction, normalization with calculation of the normalized expression values was carried out by the robust multiarray average (RMA) normalization method implemented in the “Affy” library (19). Biological annotation was obtained from the “oligo” library and combined with the normalized expression data set (20). Differential expression was calculated using linear models for microarray data from the “limma” library (21). The selection criteria for a differentially expressed genes (DEGs) based on a absolute fold change >2 and a *p* < 0.05. Technical description with raw and normalized data files were deposited in the Gene Expression Omnibus (GEO) repository at the National Center for Biotechnology Information (http://www.ncbi.nlm.nih.gov/geo/) under the GEO accession number: GSE150775.

#### Assignment of Differentially Expressed Genes to Appropriate Gene Ontology Biological Process (GO BP)

ENTREZ ids with fold change values of differentially expressed genes were subjected to gene ontology enrichment analysis using the “clusterProfiler” library (22). The analysis was performed separately for each comparison. Reference GO annotation data was obtained directly from the human annotation library “org.Hs.eg.db.” Enriched GO BP terms were visualized as a heatmap in relation to expression levels of relevant DEGs.

#### Gene Set Enrichment Analysis (GSEA)

GSEA was used to identify the level of depletion or enrichment in gene expression between two compared groups within pre-defined gene sets (GO terms, pathways). The method employs the Kolmogorov–Smirnov statistical test to determine significantly depleted or enriched gene sets (23). Analysis was carried out using the “FGSEA” Bioconductor library (24). Normalized fold change values from all of the genes were log_2_ transformed and sorted. Then using signal-to-noise ratio with 10,000 time permutation, enrichment scores (ESs) were calculated for all of predefined gene set from the Reactome database (from the Molecular Signatures Database) (25, 26). Obtained ESs were normalized by their gene set size. For each of depleted/enriched terms, the *p*-value was adjusted by FDR. Gene sets with adj. *p* < 0.05 were uploaded to Cytoscape (v. 3.7.2) to generate enrichment map with links between significantly-enriched processes (27, 28). Enriched terms were clustered and annotated using the AutoAnnotate v1.3.2 Cytoscape plugin (29). From the selected gene sets, the genes with the highest impact on the analyzed biological process that contributed the most to the enriched signal were extracted. These genes with their fold change values were visualized in Cytoscape.

### RT-qPCR Validation of Microarray Data

Real Time-PCR reactions were performed using the PrimePCR™ Custom Plates (Bio-Rad, CA, USA) and the specific synthesized primers for each gene: tissue factor pathway; jun proto-oncogene (*JUN*); histone cluster 1; H2bm (*HIST1H2BM*); arrestin domain containing 3 (*ARRCD3*); and kinesin family member 20A (*KIF20A*). The cDNA samples were analyzed for genes of interest and for the reference gene HPRT. The expression level for each target gene was calculated as −2^ΔΔct^. The reaction was performed in triplicate for the gene of interest. The result of the validation is presented in the Supplementary Figure 1.

### Inhibiting TGF-β Type I Receptor Kinase for Hormone Level Detection

To inhibit the TGF-β type I receptor (TβRI) kinase in the HAC15 cell line, we used the LY-364947 (Merck Millipore, Germany) in a final concentration of 10 μM. Prior to adropin treatment, cells were subjected to the influence of the TβRI kinase inhibitor for 1 h. Next, HAC15 cells were incubated with adropin for 24 h, using the following variants: HAC15+ DMSO (control); HAC15 + inhibitor dissolved in DMSO; HAC15+adropin+ DMSO; and HAC15+ inhibitor dissolved in DMSO +adropin. Finally, aldosterone and cortisol secretion rates were determined.

### BrdU Cytometry Analysis

After 24 h of treatment, the cells were removed from 6-well plates with 1 mM EDTA (Sigma-Aldrich, MO, USA). After detachment, cells were centrifuged and resuspended in fresh medium. Cells were counted by means of the MOXI Z automated cell counter (4 × 10^5^ cells were used for the analysis). According to manufacturer instructions, cells were fixed and permeabilised. Then, the cells were washed and stained with PerCP-Cy™5.5 Anti-BrdU antibody (51-9007682, BD Biosciences, NJ, USA). Data acquisition was performed with a multiparameter flow cytometer (CytoFLEX; Beckman Coulter, IN, USA) and results were analyzed with FlowJo software (FlowJo, LLC, OR, USA). Statistical significance was calculated on the basis of Student's *t*-test, with significance levels set at *p* < 0.05.

### RTCA Cell Proliferation Assay. Identification of Potential Adropin-Dependent Signaling Pathways Using Specific Inhibitors

To verify the effect of adropin on the proliferation rate of HAC15 cells together with the identification of the intracellular mechanism, we applied an electrical impedance-based cell proliferation assay, named Real-Time Cell Analyser (RTCA, Roche Applied Science, GmbH, Penzberg, Germany). The RTCA system detects fluctuations in electrical impedance on the integrated sensory electrodes located at the bottom of the chamber's 16-hole slide plates (E-Plate 16), which are covered by dividing cells. Electrical impedance is measured at 15-min intervals throughout the cultivation period. The main RTCA readout is the “Cell Index” –a measurable parameter corresponding to the relative change in electrical impedance depending on the rate of proliferation or apoptosis of the cultivated cells. The influence of adropin on HAC15 cell proliferation was studied in two independent experiments. In the first one it was examined whether adropin stimulates HAC15 cell proliferation through ERK1/2 and PI3/AKT dependent signaling pathway. HAC15 cells were cultivated in the following experimental groups: (1) control (2) adropin (10^−8^M) (3) PI3/AKT inhibitor–LY294002 (10^−7^M), (4) MEK1/2-dependent inhibitor of ERK1/2–U0126 (10^−7^M), (5) adropin (10^−8^M) + LY294002 (10^−7^M), (6) adropin (10^−8^M) + U0126 (10^−7^M). The second experiment examined the potential role of TGF-dependent pathway in the regulation of HAC15 cell proliferation affected by ardopin. An analogous experimental model was used here as in the experiments on the inhibition of TGF-β type I receptor kinase for hormone level detection, including following groups: (1) control, (2) adropin (10^−8^M), (3) LY-364947 (10 μM), (4) adropin (10^−8^M) + LY-364947 (10 μM).

Each experimental group was seeded in the eight E-plate wells to a final volume of 200 μl per well. The cell index was normalized (normalized cell index) at time of examined substances administration using software from the same manufacturer (RTCA Software, v. 1.2, November 2009). HAC15 cells were cultivated with examined substances per approximately 50 h. Each experiment was repeated ≥ three times.

### Normal Adrenal and Adrenocortical Carcinoma Samples

Human adrenocortical carcinoma samples were obtained from the 13 patients qualified for adrenalectomy. Normal adrenal glands (*N* = 13) were collected during the kidney transplantation procedure. After surgical removal, adrenal fragments of size 0.5 cm^3^ were immediately immersed in RNA Later Storage Solution (Sigma-Aldrich, Missouri, USA) to stabilize and protect intracellular RNA. Then samples were stored at −70°C until RNA isolation, reverse transcription and qPCR analysis were carried out. The research protocol was approved by the Bioethics Committee of PUMS (decision No. 255/15). Written consent was obtained from adrenalectomized patients as well as from kidney donors.

### Analysis of The Cancer Genome Atlas (TCGA) Dataset

Clinical description file and RNAseq counts for 92 cases of adrenocortical carcinoma were obtained from public TCGA database (30) using FireBrowse server (http://gdac.broadinstitute.org/) (31). Normalization of raw counts was done with voom algorithm from “Limma” package (21). Log transformed, normalized expression data for *ENHO* and *GPR19* gene were retrieved from the whole adrenocortical carcinoma expression dataset. The obtained data was divided in two separate populations with high and low expression of the *ENHO* and *GPR19*. The median value was taken as a cut-off point, so the expression of the examined genes above the median was assigned to the high expression group, while the samples with expression below the median was included in the low expression group. The groups were compared by Kaplan–Meier survival analysis. Survival plots with lograng *p*-value estimation were performed using “survival” R library (32) in relation to death event according to clinical description file.

## Results

### The Adropin Precursor Gene ENHO and Its Receptor (GPR19) Are Expressed in the HAC15 Adrenocortical Cell Line. Expression of the Analyzed Genes Is Relatively Stable and Not Significantly Regulated by ACTH, Forskolin, or Adropin Itself

**Figure 1:**
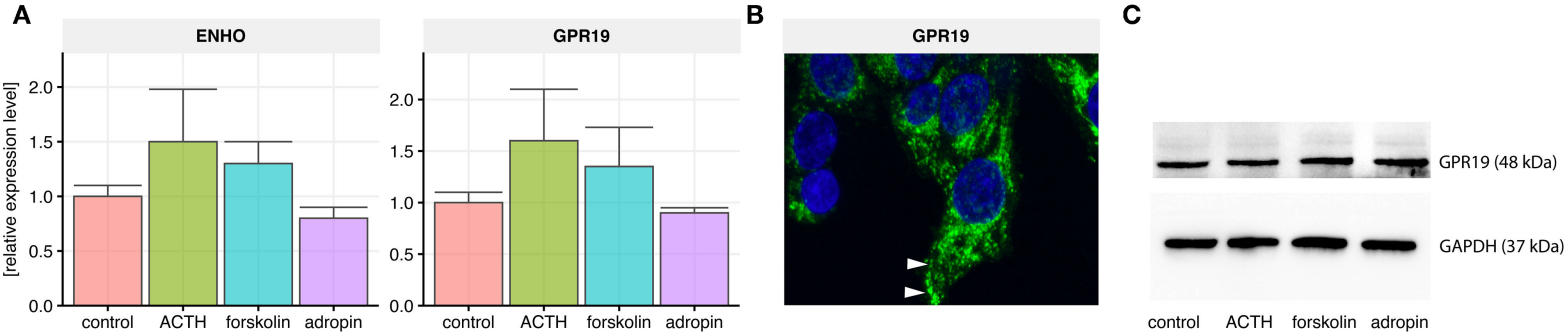
Relative expression levels of the adropin precursor gene *ENHO* and the adropin receptor *GPR19* in HAC15 cells. **(A)** Real time qPCR study in untreated (control) cells and cells incubated with ACTH, forskolin, and adropin for 24 h (*N* = 4/group). Statistical differences were determined by Mann–Whitney U non-parametric test compared to controls. Both the expression of *ENHO* and *GPR19* did not differ significantly from the control group. **(B)** GPR19 immunoreactivity in untreated HAC15 cells. The reaction was detected in cytoplasmic vesicles and cell membranes (indicated by white arrows). **(C)** Semi-quantitative analysis of GPR19 expression at protein level using western blot method.

QPCR analysis revealed expression of the adropin precursor gene (ENHO) and the adropin receptor (GPR19) in the HAC15 cell line. We also sought to determine whether this expression is regulated by the most commonly used adrenal steroidogenesis stimulators such as ACTH at a concentration of 10^−7^ M and 25 μM forskolin. Incubation for 24 h with these substances did not significantly alter the expression of the genes. We also verified the effect of adropin on the expression of the *ENHO* and *GPR19* receptor genes. Analogously to ACTH and forskolin, 24 h incubation in 10^−8^M adropin did not affect the expression of those genes (Figure 1A). We also confirmed GPR19 expression at the protein level using immunofluorescence (Figure 1B) and western blot (Figure 1C). Immuofluorescence staining revealed that GPR19 expression is mainly localized within cytoplasmic vesicles and cell membranes (Figure 1B). Semi-quantitative analysis of GPR19 expression at protein level confirmed that its expression is not regulated by the all of examined substances (Figure 1C).

### Adropin Inhibits Basal Secretion of Aldosterone and Glucocorticoids in HAC15, Y1 and PAC Cells

To analyse the effect of adropin on secretion of the main adrenocortical hormones, we utilized three different cell culture models: two adrenocortical carcinoma cell lines derived from human (HAC15), and mouse (Y1) as well as primary human adrenal carcinoma (PAC) cell culture. Approximately 70% confluent HAC15, Y1 and PAC cells seeded in 96-well plate were treated with 10^−8^ M adropin for 24 h. In all of studied cell culture models, we observed that adropin cause a significant inhibition in aldosterone and glucocorticoid secretion. In each of the analyzed cells, we noticed strong stimulation of adrenocortical hormone secretion by ACTH or forskolin, that served as a positive control. As the current research focuses mainly on the human adrenal glands, detailed results for the HAC15 cell line are presented below.

**Figure 2:**
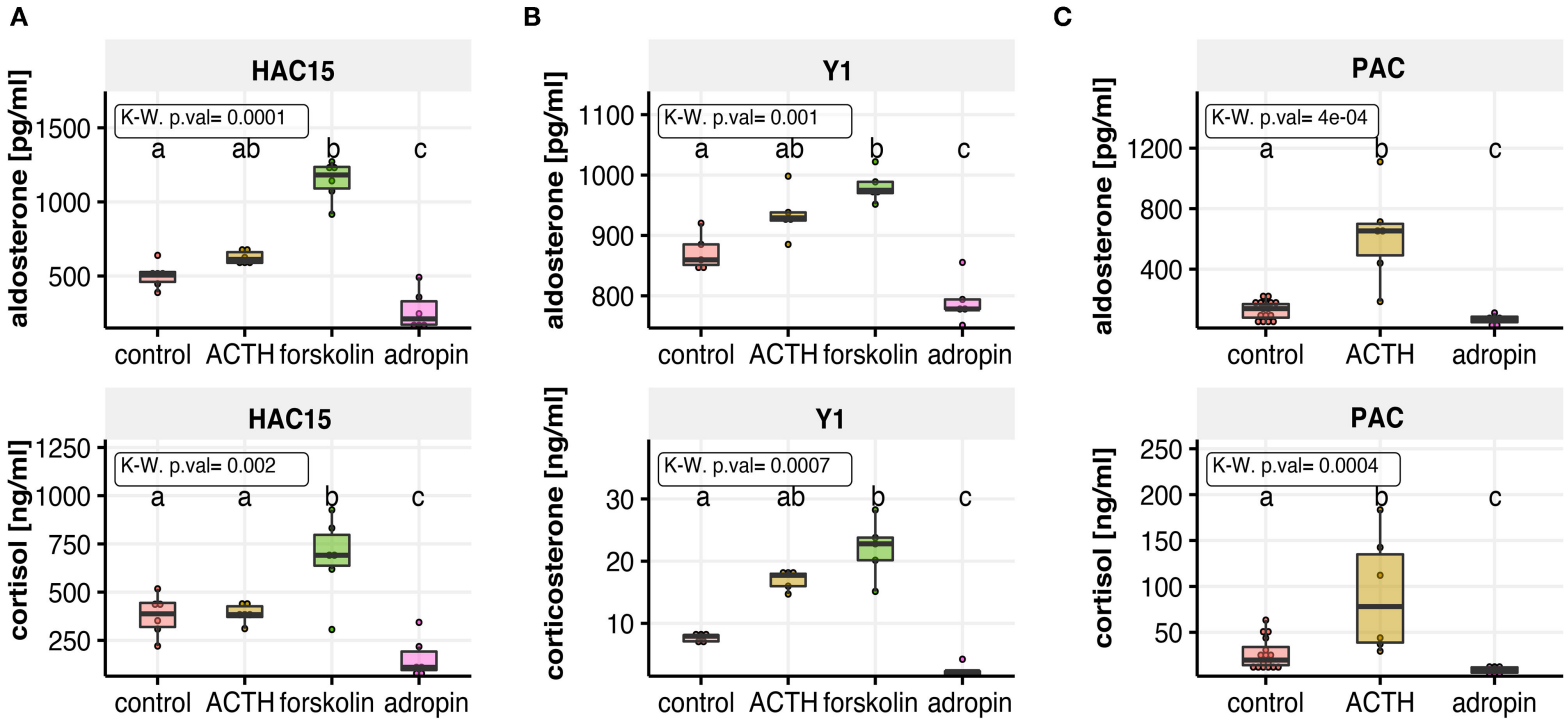
The effect of adropin on aldosterone and cortisol or corticosterone secretion in the following cell culture models: human adrenal carcinoma (HAC15) cell line **(A)**, mouse adrenocortical tumor (Y1) cell line **(B)** and primary human adrenal carcinoma (PAC) cell culture **(C)**. All of studied cells were exposed to adropin for 24 h. ACTH or forskolin treated cells were used as a positive controls. Results are presented as medians with IQR. Statistical differences were determined by Kruskal–Wallis test followed by Dunn's *post hoc* test. Groups sharing the same letter are not significantly different according to Dunn's test.

Adropin significantly reduced median aldosterone (211.4 pg/ml) and cortisol (110.21 ng/ml) secretion compared to the relevant control groups (aldosterone = 506.5 pg/ml, cortisol = 352.06 ng/ml). In both cases forskolin and ACTH were used as positive controls. Unlike H295R cells, HAC15 cells are an adrenocortical cell line with an active ACTH receptor denominated MC2R; however, the response of these cells to ACTH is relatively weak, as our results demonstrate: 24 h incubation of HAC15 cells with 10^−7^ M ACTH led to a weak stimulation of aldosterone secretion (median = 612.35 pg/ml). ACTH-stimulated cortisol secretion increased slightly (median = 383.51 ng/ml). Forskolin is a potent cAMP-dependent pathway activator, the main trigger of adrenocortical steroidogenesis and incubation of HAC15 cells with 25 μM forskolin led to rapid stimulation of both aldosterone (median = 1181.62 pg/ml) and cortisol (median = 691.37 ng/ml) secretion (Figure 2A).

### Adropin Inhibits AngII or Forskolin Stimulated Secretion of Aldosterone and Cortisol in HAC15 Cells

**Figure 3:**
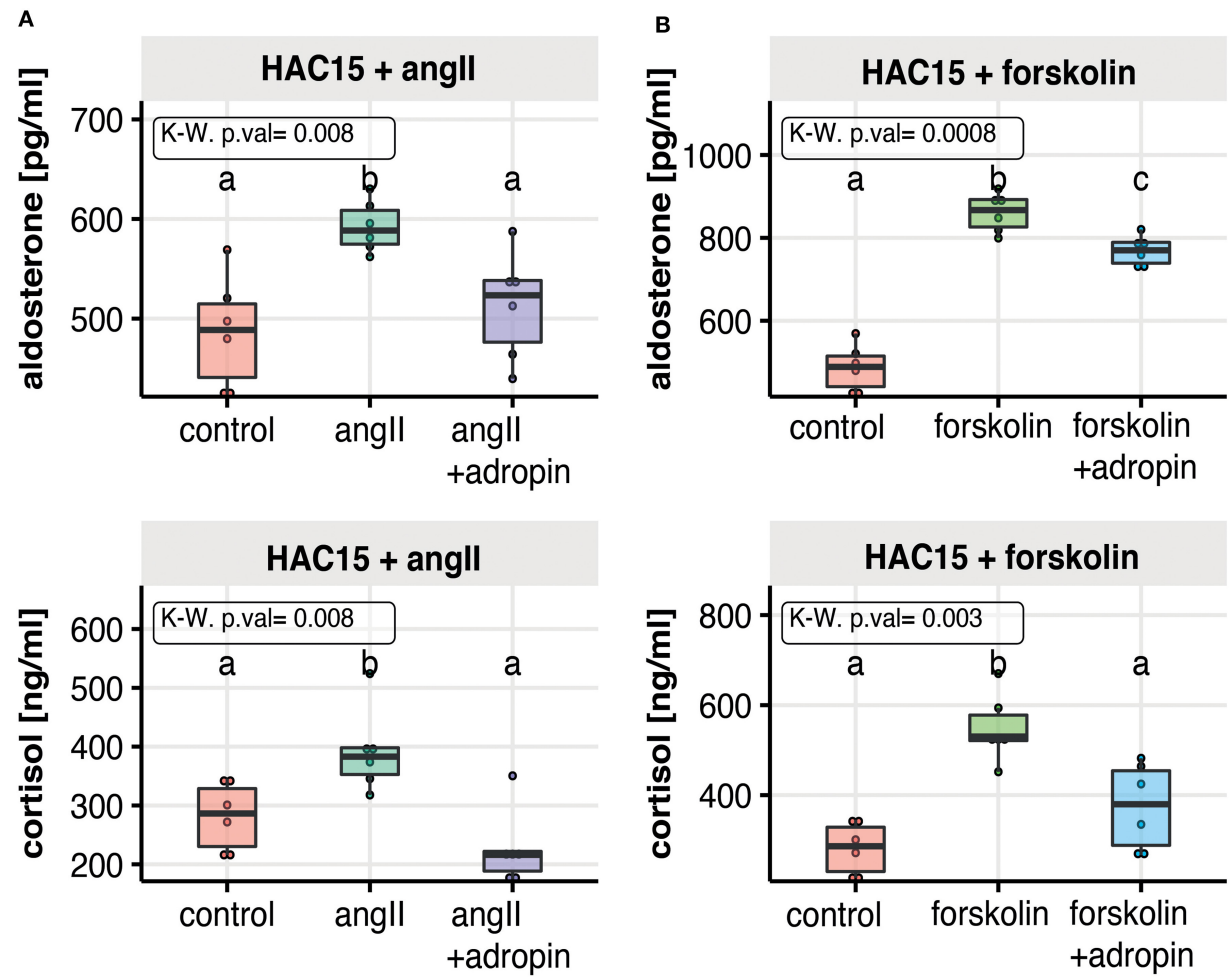
The effect of adropin on angII **(A)** or forskolin **(B)** stimulated aldosterone and cortisol secretion in the human adrenal carcinoma (HAC15) cell line exposed to adropin for 24 h. Results are presented as medians with IQR. Statistical differences were determined by Kruskal–Wallis test followed by Dunn's *post hoc* test. Groups sharing the same letter are not significantly different according to Dunn's test.

In further studies, we examined whether the inhibitory effect of adropin on adrenocortical hormones secretion could also occur after stimulation of HAC15 cells with angiotensin 2 (Figure 3A) or forskolin (Figure 3B). As expected, the secretion of the studied adrenocortical hormones was significantly increased under the influence of angII or forskolin in relation to the untreated control group. Adropin significantly reduced angII-stimulated median aldosterone (523 pg/ml) and cortisol (216 ng/ml) secretion compared to angII stimulated cells (aldosterone = 588 pg/ml, cortisol = 383 ng/ml). A similar pattern of adrenocortical hormone secretion was observed regarding to forskolin-stimulated HAC15 cells, where adropin significantly decreased forskolin-stimulated aldosterone and cortisol secretion (aldosterone = 867 pg/ml, cortisol = 380 ng/ml) in relation to forskolin-stimulated cells (aldosterone = 769 pg/ml, cortisol = 530 ng/ml).

### Inhibitory Effect of Adropin on Adrenocortical Steroidogenesis Occurs by Decreasing the Expression of Rate-Limiting Genes for the Initial Steps of Steroid Biosynthesis

**Figure 4:**
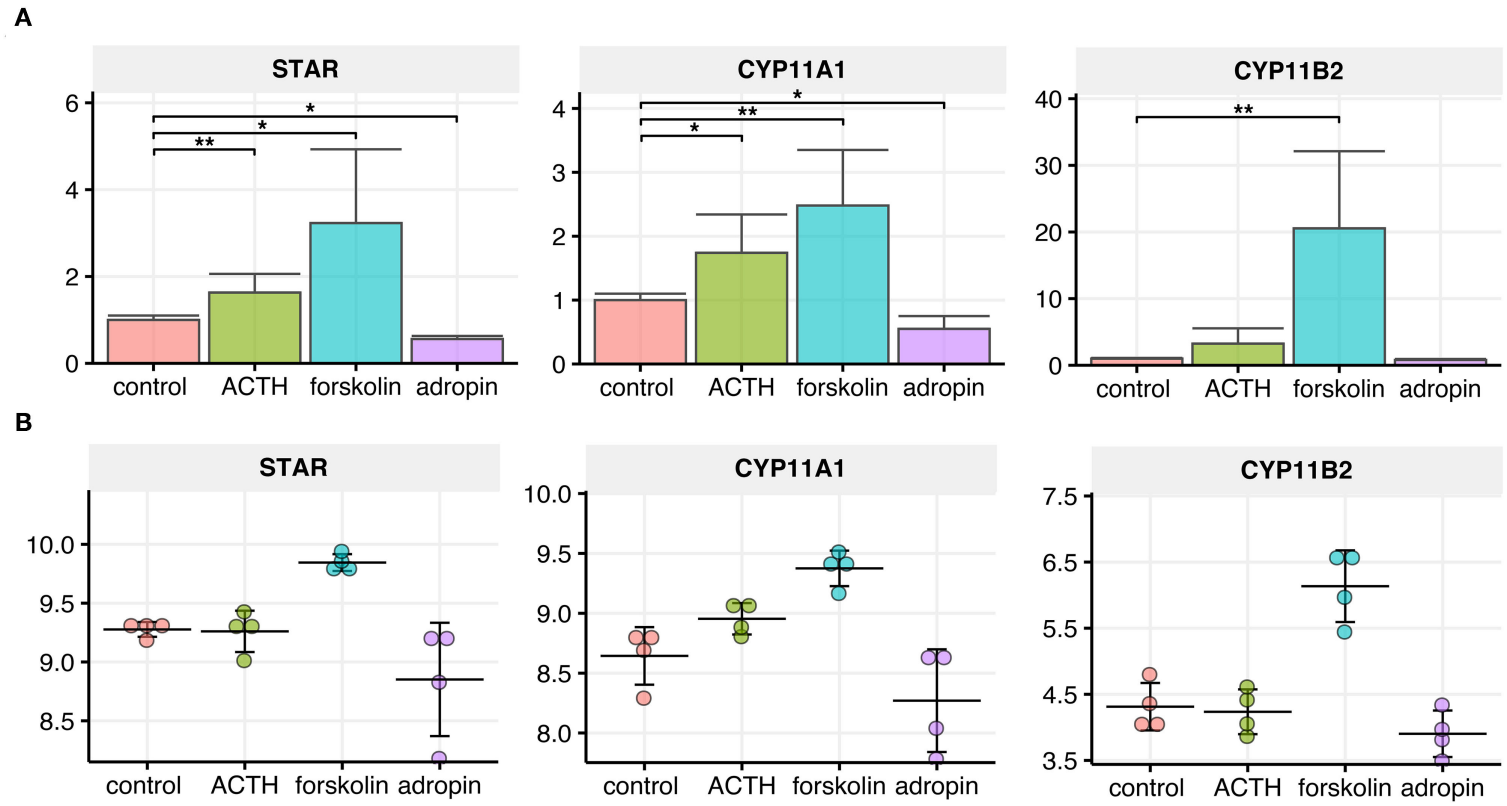
Relative expression levels of *CYP11A1, CYP11B2* and *StAR* genes in the HAC15 cell line after 24 h incubation with ACTH, forskolin, and adropin vs. controls (untreated cells). **(A)** Real time QPCR study (*N* = 4/group). Statistical differences were determined by the Mann–Whitney U non-parametric test compared to controls. *p < 0.05; **p < 0.01. **(B)** Normalized expression values extracted from the microarray data set (*N* = 4/group).

The steroidogenic acute regulatory protein (StAR) and side-chain cleavage enzyme (CYP11A1) catalyze the first, rate-limiting step of steroidogenesis. StAR enables cholesterol translocation into the inner mitochondrial membrane (IMM), where CYP11A1 transforms cholesterol to pregnenolone. To assess the relative changes in *StAR* and *CYP11A1* gene expression following adropin treatment, we performed relative QPCR using specific primers. Additionally, we extracted normalized data from a microarray study of the genes. According to the QPCR, 24 h incubation of HAC15 cells with adropin resulted in a statistically significant decrease in expression of *StAR* and the *CYP11A1* gene (44 and 45% from baseline values, respectively) (Figure 4). *StAR* and *CYP11A1* gene expression presented a similar profile in the microarray study, although statistically insignificant compared to controls. As expected, forskolin strongly stimulated the expression of *StAR* and *CYP11A1*, which were, respectively, 3.23 and 2.48 times higher than controls. Similarly, ACTH was also stimulative, but only with the QPCR method, where ACTH increased *StAR* expression by 1.63 and *CYP11A1* expression by 1.74. Adropin did not affect expression of the *CYP11B2* gene encoding aldosterone synthase; only forskolin induced a significant stimulation of *CYP11B2* expression (20.54-fold increase).

**Figure 5:**
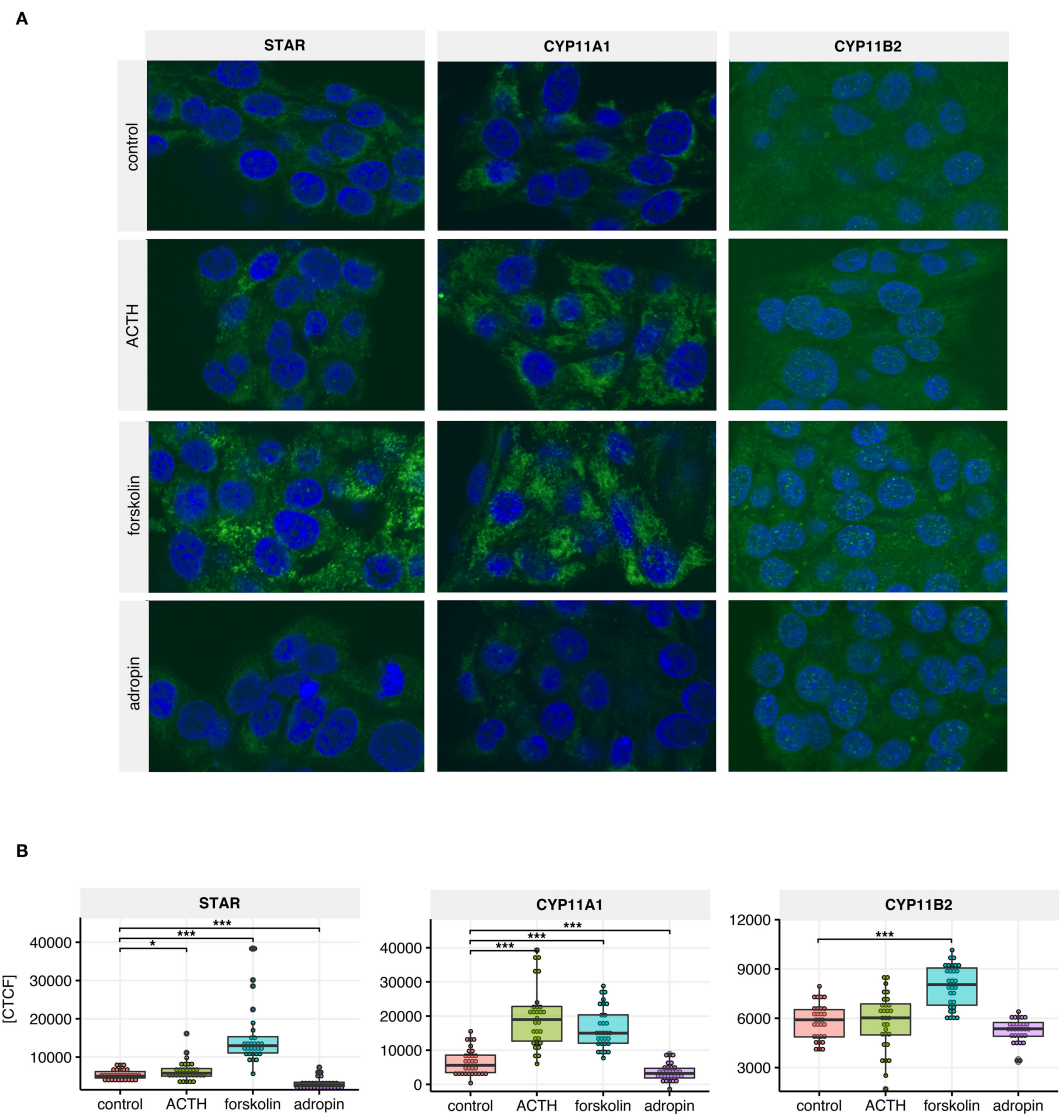
StAR, CYP11A1, CYP11B2 immunoreactivity in untreated HAC15 cells (controls) and cells incubated with ACTH, forskolin and adropin for 24 h. **(A)** Representative images of the HAC15 cells from each group, where the positive reaction of antibody binding was visualized in green, while the cell nuclei were stained in blue with DAPI. **(B)** Densitometric analysis of the fluorescence intensity (*N* =3 0/group). Statistical differences were determined by the Mann–Whitney U non-parametric test compared to controls. *p < 0.05; ***p < 0.001. CTCF indicates corrected total cell fluorescence.

Next, we performed semi-quantitative analysis of StAR, CYP11A1 and CYP11B2 expression at the protein level using immunofluorescence. The use of identical experimental conditions and the same laser power for fluorochrome excitation allowed us to evaluate signal intensity and consequently to perform semi-quantitative analysis of StAR, CYP11A1 and CYP11B2 expressed in HAC15 cells subjected to 24 h ACTH, forskolin, or adropin stimulation. Representative images and a summary graph with statistical analyses are presented in Figure 5. In general, the expression profiles of StAR, CYP11A1 and CYP11B2 proteins correspond to the expression profile of their respective genes. Our data show that 24 h of adropin incubation with HAC15 cells led to a 2.88-fold decrease in StAR protein expression vs. the control group (median CTCF 1,447 vs. 4,177, respectively). Adropin also inhibited expression of the CYP11A1 protein, leading to 1.49-fold decrease in CYP11A1 expression vs. controls (median CTCF, 4,264 vs. 6,357). Adropin did not significantly alter CYP11B2 expression vs. controls (median CTCF = 5,083 vs. 5,880, respectively). Incubation of HAC15 cells with ACTH led to 1.23-fold increase in StAR protein expression (median CTCF = 5,169) and 2.8-fold increase in CYP11A1 (median CTCF = 17,801), without a significant effect on CYP11B2 protein expression (median CTCF = 6,010). Forskolin caused a significant increase in expression of StAR, CYP11A1 and CYP11B2 proteins, leading to a 3.2-, 2.26- and 1.49-fold increase in their expression, respectively (median CTCF values: StAR = 13,648, CYP11A1 = 14,377, and CYP12B2 = 8,756).

### Adropin Moderately Alters the Transcriptomic Profile of HAC15 Cells

**Figure 6:**
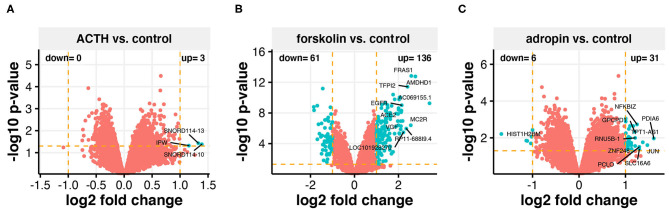
Total gene expression profile of the HAC15 cell line after 24 h incubation with ACTH **(A)**, forskolin **(B)**, and adropin **(C)** compared to control (untreated) cells. Each dot represents a mean value of the single gene expression (*n* = 4/group). Dotted lines indicate cut-off values (two-fold change in expression and *p* < 0.05 with FDR correction). The red dots represent the genes below the cut-off limit. The turquoise color marks upregulated and downregulated genes. Ten genes with the highest differences within fold change values were labeled with the appropriate gene symbols.

The Affymterix Gene Chip Human Genome U219 Array Strips used in the current study allowed for the simultaneous examination of gene expression of 19,285 human transcripts. The transcriptome study was performed 24 h after adding ACTH, forskolin, or adropin to the culture medium. The transcriptomic profile was compared to the group of untreated cells (controls). The general profile of transcriptome changes is shown as a volcano plot (Figure 6). We assumed the following selection criteria for DEG: an expression |fold difference| (absolute value)> 2 and *p* ≤ 0.05. According to these criteria, only three genes were significantly upregulated after ACTH treatment, while 136 genes were upregulated and 61 downregulated by forskolin. Adropin caused a decrease in the expression of 6 genes and an increase in 31 genes. Genes with significant changes and the highest fold values are marked by their symbols on the volcano plot graphs. Adropin significantly altered expression in the following genes: histone cluster 1; H2BM (*HIST1H2BM*; fold = −3.19); nuclear factor of kappa light polypeptide gene enhancer in B-cells inhibitor; zeta (*NFKBIZ*, fold = 2.38); glycerol phosphocholine phosphodiesterase GDE1 homolog (*GPCPD1*, fold = 2.3); protein disulfide isomerase family A, member 6 (*PDIA6*, fold = 3.05); TPT1 antisense RNA 1 (*TPT1-AS1*, fold = 2.59); RNA, U5B small nuclear 1 (*RNU5B-1*, fold = 2.31); zinc finger protein 248 (*ZNF248*, fold = 2.31); jun proto-oncogene (*JUN*, fold = 2.77); piccolo presynaptic cytomatrix protein (*PCLO*, fold = 2.46); and solute carrier family 16, member 6 (*SLC16A6*, fold = 2.46)

**Figure 7:**
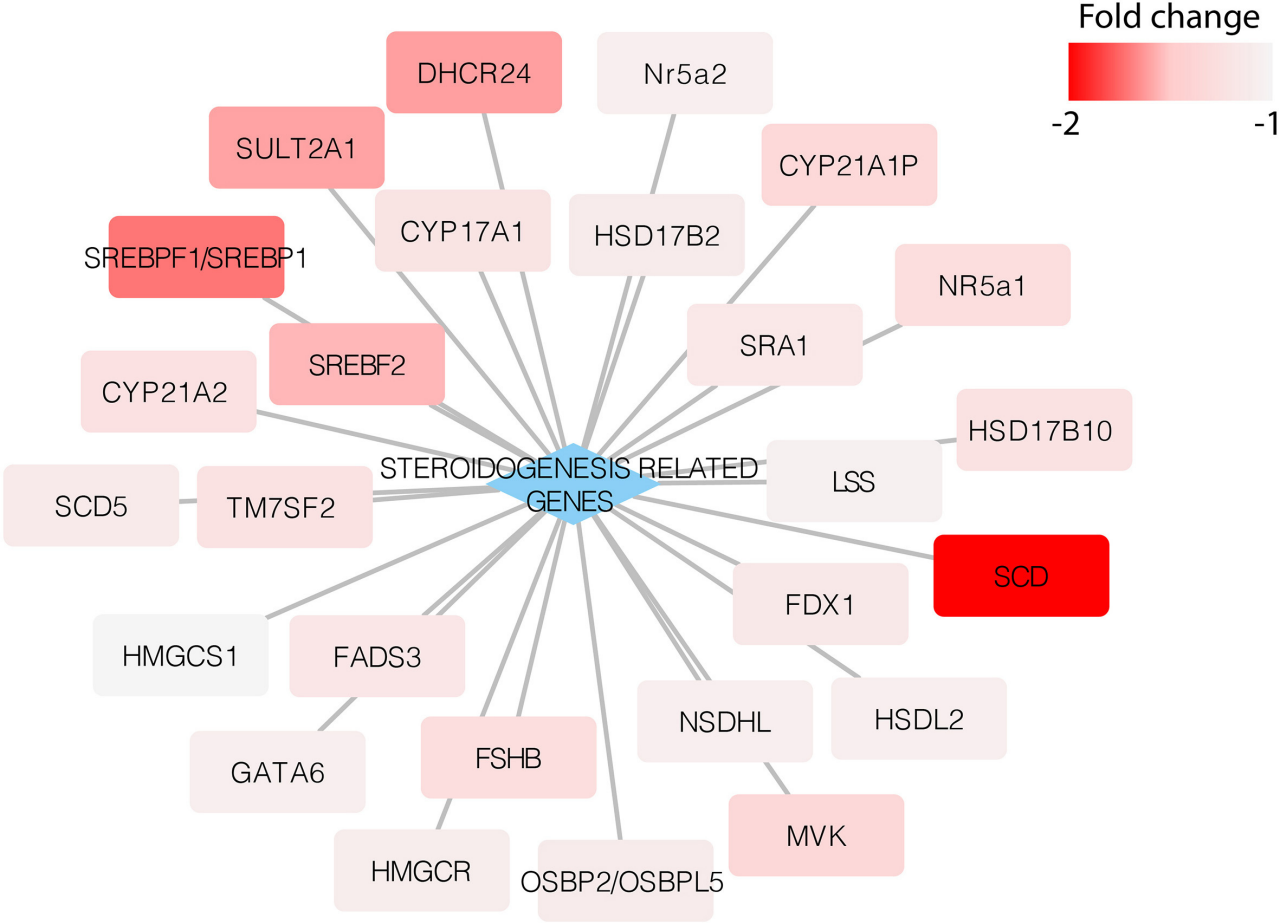
Expression of steroidogenesis-related genes in the HAC15 cell line after 24 h incubation with adropin compared to control (untreated) cells. Appropriate fold change values are marked in red.

After studying the steroidogenesis-related genes, we found that adropin decreased the expression of these genes. Although the fold values in some of the genes were below the cut-off threshold, there was an evident trend showing the capacity of adropin to inhibit steroidogenesis-related gene expression (Figure 7).

### Adropin Exerts a Significant Effect on the Genes Involved in the TGF Beta Signaling Pathway

**Figure 8:**
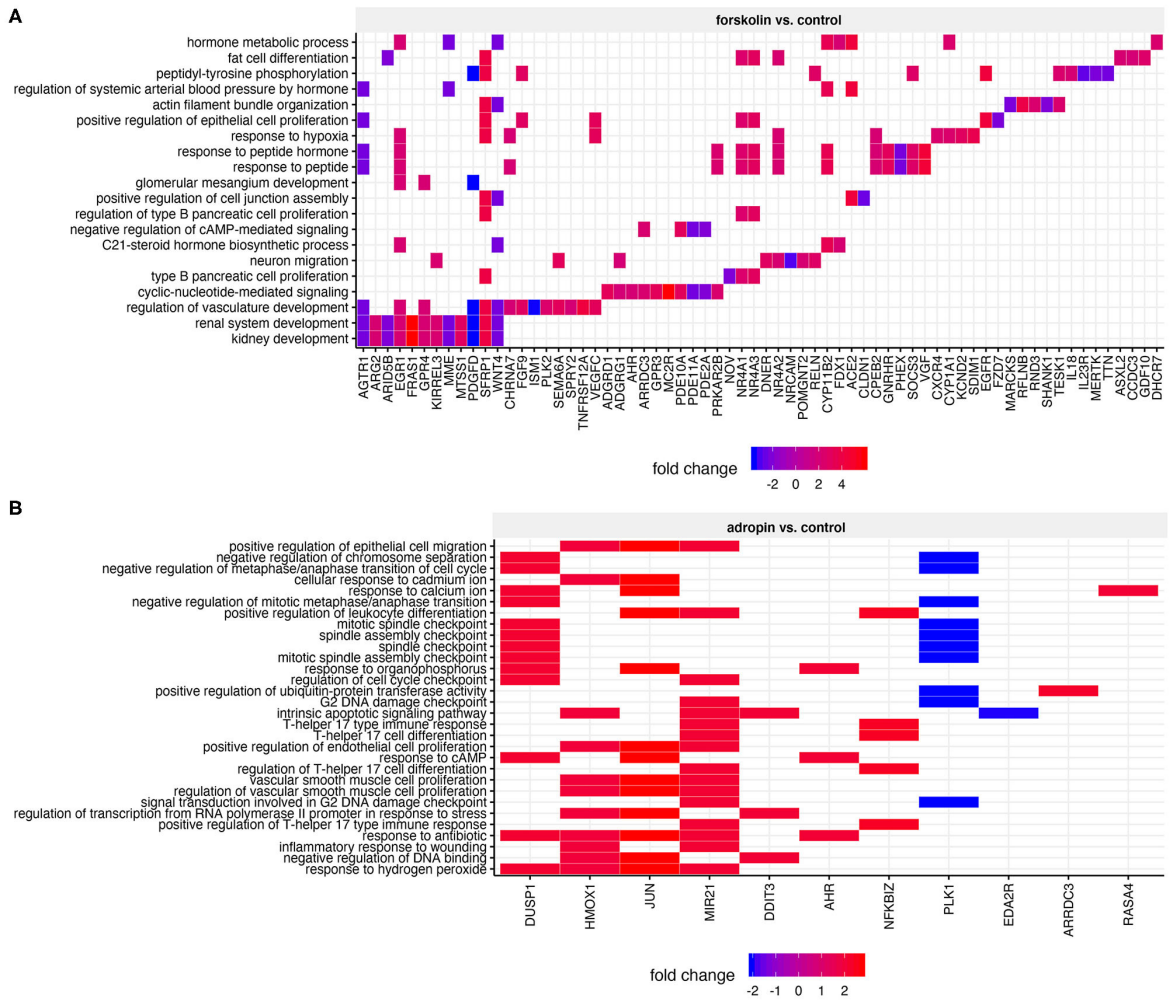
Heatmap of overrepresented gene sets from GO BP (gene ontology–biological process) database, regulated by forskolin **(A)** and adropin **(B)** compared to the control group. The graphs show only the GO groups above the established cut-off criteria (adjusted *p* < 0.05). Fold change values for each DEG were mapped with the appropriate color. Gene symbols of DEGs are shown on the x scale.

To determine the biological processes most likely to be regulated by adropin, we analyzed enrichment in the relevant ontological groups from the GO BP database. We performed a similar analysis for forskolin. Whole sets of differentially expressed genes (197 for forskolin vs. controls; 37 for adropin vs. controls) were subjected to gene ontology enrichment analysis using ClusterProfiler. The result of this analysis is shown as a heatmap (Figure 8). The results show that adropin activated 30 different biological processes, most of which (*N* = 15) were related to processes involved in cell cycle regulation, including the following: “signal transduction involved in G2 DNA damage checkpoint”; “positive regulation of endothelial cell proliferation”; “G2 DNA damage checkpoint” “regulation of cell cycle checkpoint”; “mitotic spindle assembly checkpoint” “negative regulation of mitotic metaphase/anaphase transition”; “negative regulation of metaphase/anaphase transition of cell cycle”; “negative regulation of chromosome separation.” Another significant cluster of gene ontology terms concerned the regulation of immune response process: “positive regulation of leukocyte differentiation”; “inflammatory response to wounding”; “positive regulation of T-helper 17 type immune response”; “regulation of T-helper 17 cell differentiation”; “T-helper 17 type immune response”; “T-helper 17 cell differentiation.”

**Figure 9:**
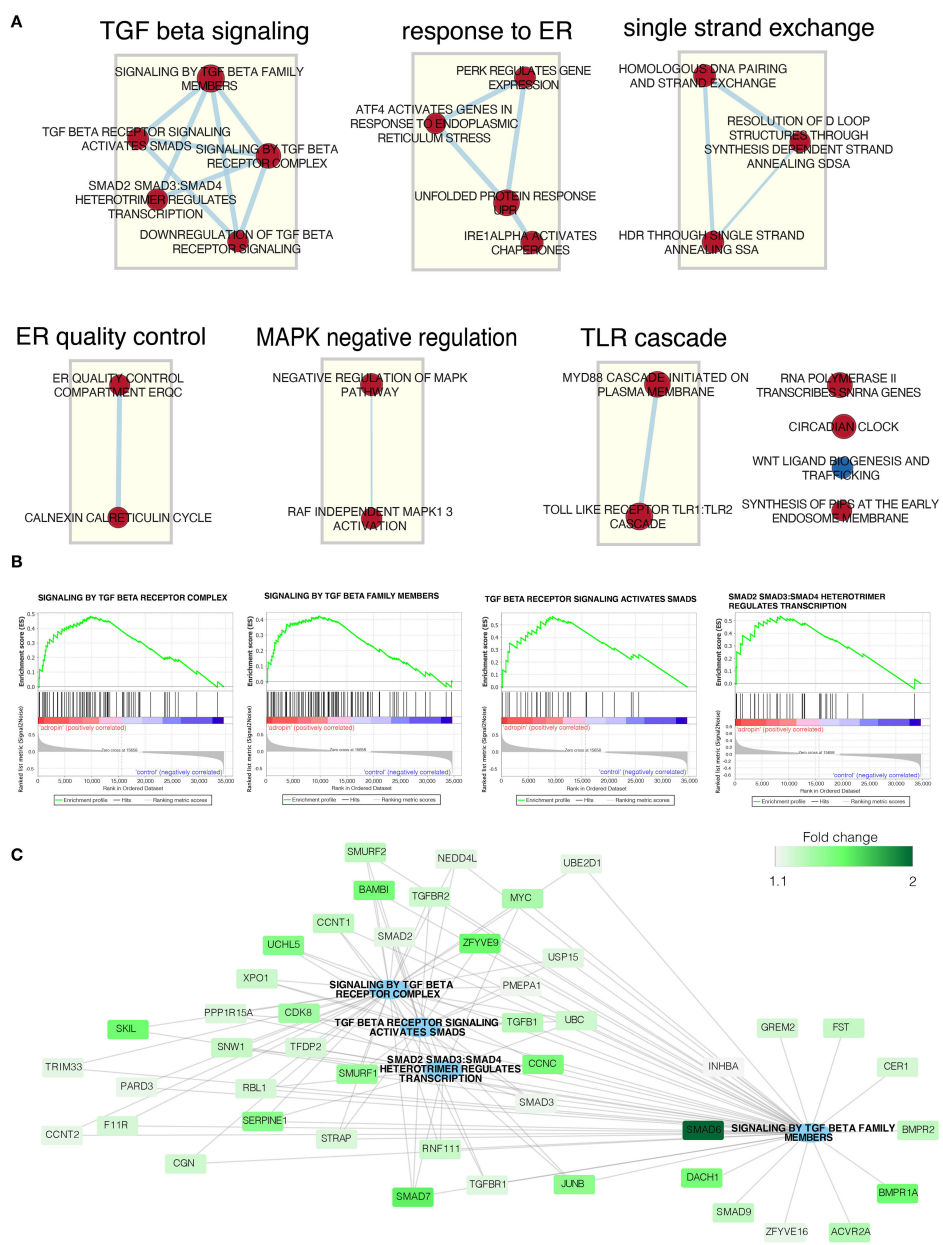
Gene set enrichment analysis affected by adropin compared to control group. **(A)** Enrichment map of gene sets regulated by adropin (red = activated, blue = inhibited). The thickness of the connecting line corresponds to the level of gene matching to individual terms. **(B)** Enrichment plot of: “Signaling by TGF beta receptor complex,” “Signaling by TGF beta family members,” “TGF beta receptor signaling activated by SMADs,” “SMAD2 SMAD3:SMAD4 heterotrimer regulates transcription.” Each of the graphs shows the positive value of the enrichment score (ES) indicating activation of genes forming these processes under the influence of adropin. **(C)** Interaction network between genes with the highest impact on the biological processes analyzed. Fold change values for individual genes are marked by color scale.

The above analysis of ontological groups was carried out in the relatively small group of adropin-regulated genes that met the assumed cut-off criteria (fold > 2, *p* < 0.05), which explains why the ontological groups presented in Figure 8 consist of a small number of genes. This suggests that the transcriptome profile affected by 24 h incubation of HAC15 cells with adropin may be regulated in a limited manner, as evidenced by the fact that most genes did not pass the cut-off criteria. Therefore, we decided to rely on GSEA based on the ranking of all genes regardless of the assumed cut-off values. In this analysis, the fold change values of all genes were log_2_ transformed and ranked according to their logFC value. Next, these values were used for a 10,000 time permutation test to calculate the enrichment score (ES) within predefined gene sets from the Reactome database. Enrichment scores were normalized by their gene set size and presented as a normalized enrichment score (NES) in which positive NES values indicate that the process is stimulated by adropin. Gene sets with adjusted *p* < 0.05 were exported to Cytoscape software to generate links between significantly enriched processes (Enrichment Map) (Figure 9A). The largest cluster of enriched terms were related to “TGF beta signaling,” for which we found five significant enrichment terms: “Signaling by TGF beta family members” (NES = 1.81, adj. *p* = 0.042); “TGF beta receptor signaling activates SMADs” (NES = 1.86, adj. *p* = 0.036); “Signaling by TGF beta receptor complex” (NES = 1.92, adj. *p* = 0.037); “SMAD2 SMAD3:SMAD4 heterotrimer regulates transcription” (NES = 1.83, adj. *p* = 0.041); and “Downregulation of TGF beta receptor signaling” (NES = 1.95, adj. *p* = 0.036). Interaction network between genes with the highest impact on “TGF beta signaling” enriched terms are presented in Figure 9B. The expression of all presented genes increased under the influence of adropin stimulation, suggesting that this peptide affects physiological function of HAC15 cells via a TGF beta-dependent pathway.

### Adropin Inhibits Adrenocortical Steroidogenesis via TGF Beta Mediated Pathway

**Figure 10:**
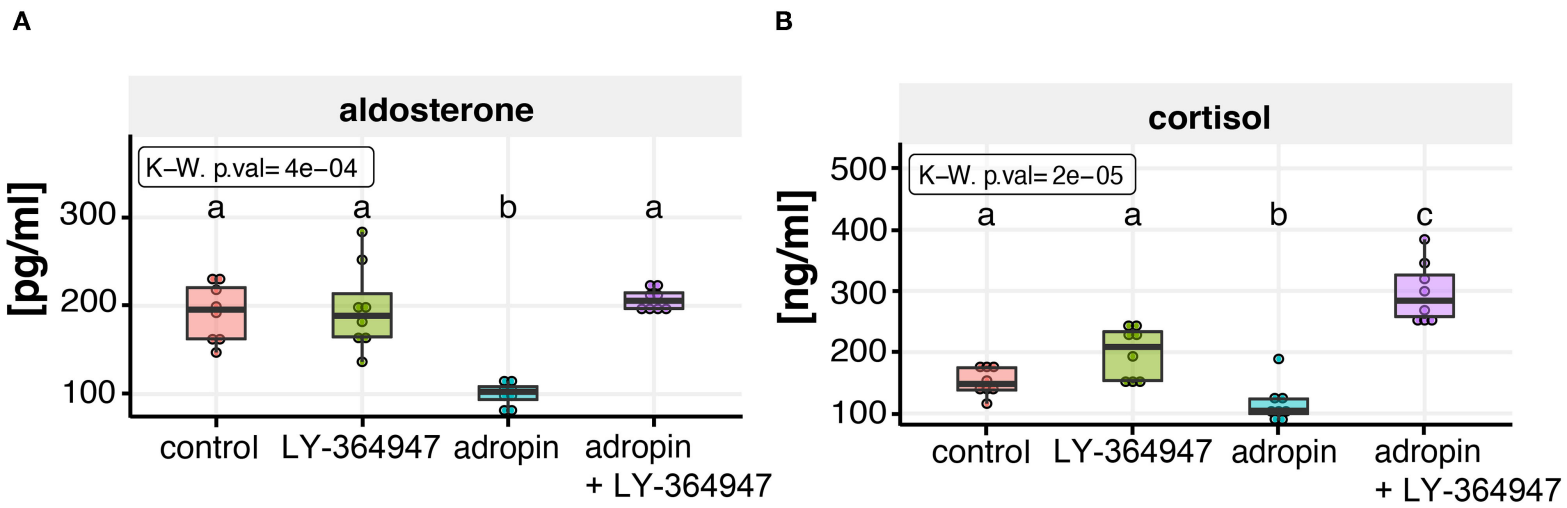
Effect of TGF beta signaling inhibitor (LY-364947) on adropin dependent aldosterone **(A)** and cortisol **(B)** inhibition in HAC15 cells. Approximately 70% confluent HAC15 cells seeded in 96-well plates were incubated with LY-364947 for 1 h prior to subsequent 24 h adropin treatment. Aldosterone and cortisol secretion rates were statistically evaluated by the Kruskal–Wallis test followed by Dunn's *post hoc* test. Groups sharing the same letter are not significantly different according to Dunn's test.

The experiment was conducted on 70% confluent HAC15 cells seeded in 96-well plates. In order to inhibit the TGF beta–dependent cell response, the TβRI kinase inhibitor was given to appropriate groups (I) and then the HAC15 cells were incubated for 1 h. Next, adropin was administered. The results are shown in Figure 10. The inhibitor itself had no significant effect on median aldosterone (188.68 pg/ml) and cortisol (208.54 ng/ml) secretion compared to controls (aldosterone =195.59 pg/ml, cortisol = 141.49 ng/ml). As noted above, adropin significantly reduced median aldosterone (102.6 pg/ml) and cortisol secretion (104.4 ng/ml). However, in the group in which TGF-beta signaling was inhibited by adropin administration, this effect was either completely absent for aldosterone (205.68 pg/ml) or it resulted an increase of cortisol secretion (283.96 ng/ml). These results confirm that adropin has the capacity to inhibit adrenocortical steroidogenesis of HAC15 cells via the TGF-β mediated pathway.

### Adropin Stimulates the Proliferative Activity of HAC15 Cells via ERK1/2 and AKT, Independently of TGF Beta Mediated Pathway

**Figure 11:**
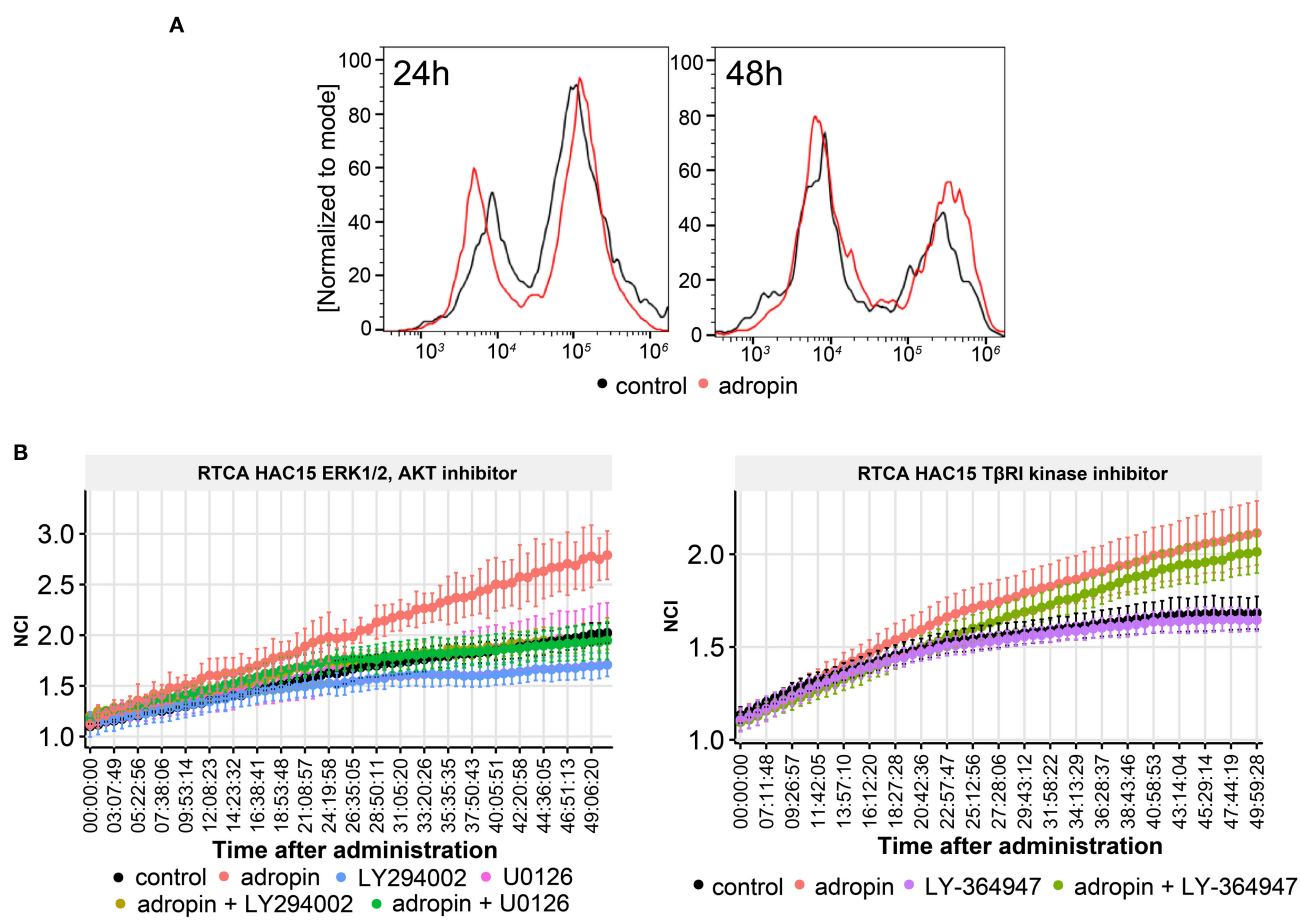
The role of adropin in HAC15 cell proliferation. **(A)** Flow cytometry study on the contribution of adropin (10^−8^ M concentration) in stimulation of HAC15 cell proliferation, measured at 24 and 48 h after peptide administration. **(B)** Effect of ERK 1/2, AKT and TBRI kinase inhibitors on adropin-induced HAC15 cell proliferation using xCelligence Real-Time Cell Analyzer–RTCA **(B)**. The RTCA chart presents mean normalized cell index (NCI) +-SD.

The effect of 10^−8^M adropin on the proliferation of HAC15 cells was determined by two independent methods. First, we applied flow cytometry method involving cell labeling with BrdU (Figure 11A). The measurement was made –at 24 and 48 h after peptide administration. At 24 h post-adropin administration, we observed no significant changes in the number of HAC15 cells vs. the untreated control group; however, after 48 h, the proliferation rate of HAC15 cells incubated with adropin had increased significantly (Figure 11A). The second method of proliferation rate testing based on impedance measurement of proliferating cells using the xCelligence (RTCA). This method enables continuous, real-time proliferation assessment, measured in 15-min intervals. Within 50 h from adropin administration we observed again its stimulatory effect on proliferation (Figure 11B) To verify whether the adropin stimulatory effect on HAC15 cells proliferation depends on ERK1/2 and AKT activation, we perform RTCA analysis in the presence of pharmacological MEK1/2-dependent inhibitor of ERK1/2 (U0126) and PI3/AKT inhibitor (LY294002). We have also investigated the potential role of TGF beta-dependent pathway in HAC15 cells proliferation using LY-364947 inhibitor (Figure 11B). In the presence of ERK1/2 and PI3/AKT inhibitors, adropin induced HAC15 cells proliferation was completely inhibited (adropin + U0126 and adropin + LY294002 in relation to adropin group on Figure 11B left side). On the other hand, the presence of LY-364947 in the culture medium did not significantly suppress the stimulating effects of adropin (adropin + LY-364947 in relation to adropin group on Figure 11B right side). These data suggest that adropin stimulate HAC15 cells via ERK1/2 and AKT dependent mechanism independently of TGF beta mediated pathway.

### GPR19 Expression Is Elevated in Human Adrenocortical Carcinoma. High Level of GPR19 Could Constitute a Negative Prognostic Factor of Disease Progression

**Figure 12:**
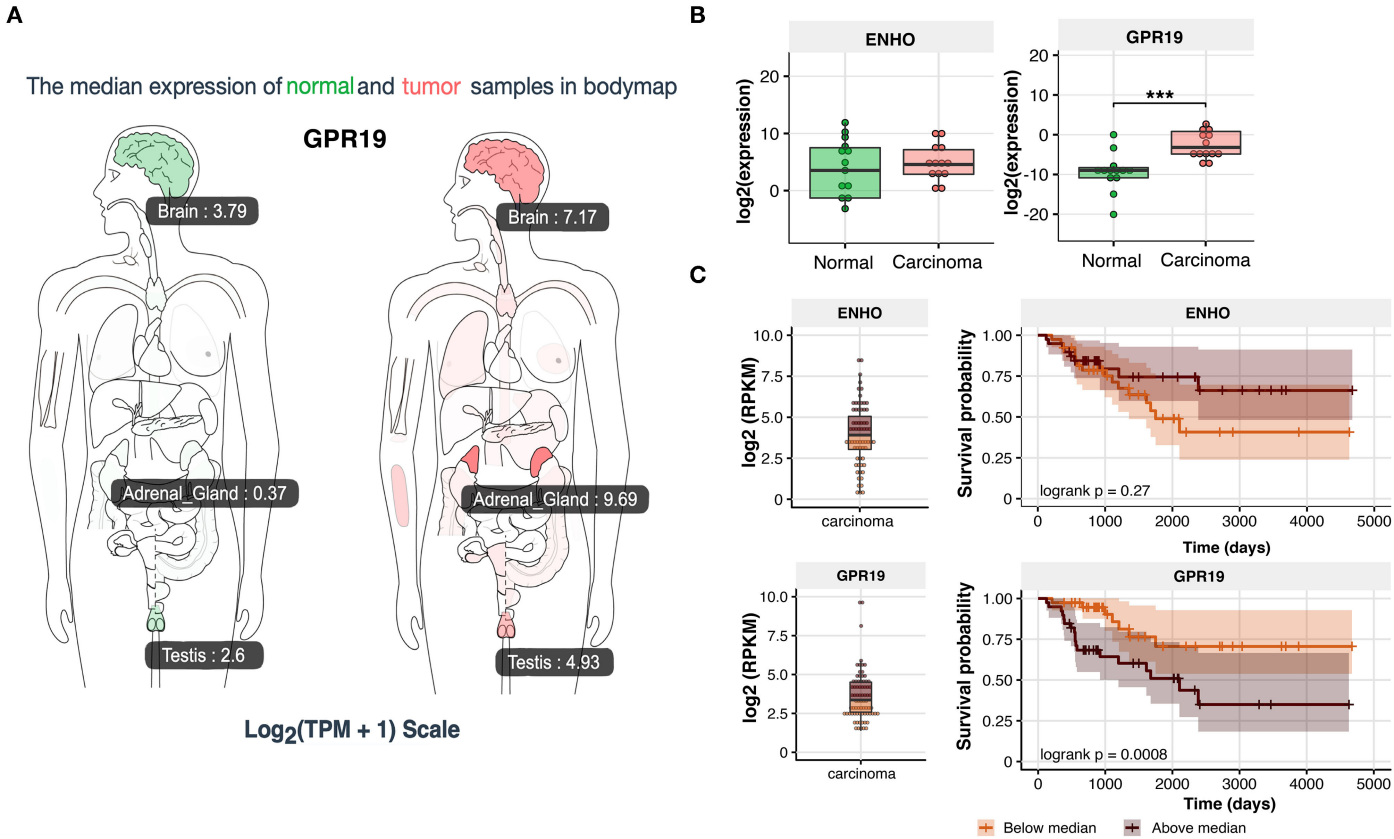
Expression of *ENHO* and *GPR19* in clinical samples. **(A)** Profile of *GPR19* expression in normal and tumor samples from GEPIA (Gene Expression Profiling Interactive Analysis) database. **(B)** Expression of *ENHO* and *GPR19* in normal and tumor samples of human adrenal gland. **(C)** Kaplan–Meier survival analysis of 92 cases of adrenocortical carcinoma obtained from TCGA database. Expression data for *ENHO* and *GPR19* were divided into group with high (above median) and low expression (bellow median). Statistical differences were determined by the Mann–Whitney U non-parametric test compared to normal adrenals. ***p < 0.001.

Due to the significant stimulatory role of the adropin-GPR19 system in the proliferation of human derived adrenocortical carcinoma cell line (HAC15), mediated by ERK1/2 and AKT dependent mechanism, we undertook the analysis regarding the clinical significance of the obtained results. According GEPIA (Gene Expression Profiling Interactive Analysis) (33) web server for cancer and normal gene expression profiling based on RNA-seq data, expression of *GPR19* in normal human organs is the highest in brain and testis. These observations are consistent with the data deposited in the NCBI database (https://www.ncbi.nlm.nih.gov/gene/2842). According to GEPIA and NCBI data, expression of *GPR19* in normal adrenal gland is relatively low, but it' s strongly rising in adrenal carcinoma (0.37 in normal adrenal vs. 9.69 in adrenal carcinoma according to GEPIA) (Figure 12A). Expression analysis of our normal adrenal samples in relation to adrenocortical carcinoma (*N* = 13/group) showed that the expression of the adropin precursor gene–*ENHO* was not regulated in the study groups (Figure 12B). Expression of *GPR19* was significantly increased in adrenocortical carcinoma, which is in line with previously reported GEIPA data. According Kaplan–Meier survival analysis of data received from TCGA, expression of *ENHO* gene do not have a significant impact on the survival profile of patients with adrenocortical carcinoma (logrank *p* = 0.27) (Figure 12C), however elevated expression of *GPR19* leads to a statistically significant decrease in survival (logrank *p* = 0.0008), suggesting that high expression of *GPR19* could constitute a negative prognostic factor of disease progression.

## Discussion

Adropin is a newly identified metabolic hormone expressed inter alia in the liver and brain. It plays a role in mechanisms related to increased adiposity, insulin resistance, as well as glucose and lipid metabolism (1). Adropin is a secretory signal peptide which has been shown to alter whole body glucose and lipid metabolism after administration in mice and rats, and it also activates signaling pathways involved in glucose and lipid metabolism in mammalian cell lines (34, 35).

To verify our hypothesis that adropin plays an important role in adrenocortical hormone biosynthesis, we selected the human adrenal cell model based on the HAC15 cell line because HAC15 cells secrete mineralocorticoids, glucocorticosteroids, and adrenal androgens. While numerous experiments have been carried out on NCI-H295 cells, our *in vitro* research model with HAC15 cells seems to be more valuable because, unlike NCI-H295 cells, HAC15 adrenocortical cells react to some extent to corticotropin stimulation and also have good forskolin response (36, 37).

The current study has several objectives: (1) to determine the impact of adropin on the proliferation rate of HAC15 adrenocortical cells with analysis of ERK1/2, AKT signaling pathway contribution in this process, (2) to evaluate the role of the adropin in the regulation of primary physiological function in adrenocortical cells (i.e., mineralocorticoid and glucocorticoid secretion), (3) to examine the response of HAC15 adrenocortical cells to treatment with adropin, forskolin, and ACTH at the gene and protein level, (4) to assess how administration of adropin changes the expression of key genes involved in steroidogenesis, and (5) to evaluate clinical significance of the obtained results by analysis of *ENHO* and *GPR19* gene expression in human normal adrenals in relation to adrenocortical carcinoma.

To our knowledge, the current study is the first to examine the direct effect of adropin on the regulation of adrenocortical steroidogenesis. We have shown that exposure to adropin significantly inhibits the basal and angII or forskolin stimulated secretion of aldosterone and cortisol by HAC15 cells. Adropin reduced also the secretion of adrenocortical hormones in Y1–mouse adrenocortical carcinoma cell lines as well as in primary human adrenal carcinoma cell culture, proving its universal mechanism of inhibiting the secretion of adrenal hormones from tumor derived adrenocortical cells. Thus, adropin belongs to the group of biologically active peptides that directly inhibit adrenocortical steroidogenesis. Other peptides in this group include bursal anti-steroidogenic peptide (BASP) (38); atrial natriuretic peptide (ANP) (39); B-type natriuretic peptide (BNP) (40); and thyrotropin (TRH) (41).

We also proved that adropin-mediated adrenal steroidogenesis inhibition occurs by suppressing expression of the StAR protein and CYP11A1 gene, which are involved in the initial stages of steroidogenesis. The steroidogenic acute regulatory protein (StAR) and side-chain cleavage enzyme (CYP11A1) catalyze the first and rate-limiting step of steroidogenesis. StAR enables cholesterol translocation into the inner mitochondrial membrane (IMM), where CYP11A1 transforms cholesterol to pregnenolone (42).

We also evaluated the molecular aspects of inhibition of steroidogenesis by adropin in HAC15 cells through global gene expression analysis using a microarray approach. HAC15 cells exposed to adropin showed altered expression of genes that regulate biological processes such as cell cycle regulation and immune response; however, the overall expression profile was characterized by a relatively weak modulation, which is reflected by a low number of genes above the cut-off values in the volcano plot. Further analysis with GSEA revealed that adropin induces the expression of a gene cluster associated with the TGF-β signaling pathway. This observation allowed us to suggest that adropin-mediated inhibition of steroidogenesis may occur through the activation of the TGF-β signaling pathway. Accordingly, TGFβ appears to be a negative regulator of adrenocortical steroidogenesis. In this context, other studies have shown that TGFβ inhibits adrenal hormone biosynthesis in sheep (43) and bovine primary cultures of adrenocortical cells (44). Furthermore, Liakos et al. observed that TGFβ1 significantly inhibits forskolin-induced steroid 11β-hydroxylase activity and CYP11B1 mRNA levels, as well as angiotensin II-induced aldosterone synthase activity and CYP11B2 mRNA levels in the human adrenocortical tumor cell line NCI-H295R (45). Another study demonstrated that TGF-β1 inhibits progesterone and estradiol production by trophoblast cells; however, these inhibitory effects were diminished if the cholesterol transport and aromatization steps were bypassed, suggesting that the action of TGF-β1 on steroid production is exerted at the level of cholesterol transport and aromatase activity (46). Consistent with these reports, other studies have confirmed that TGF-β1 downregulates StAR expression and decreases progesterone production in cultured human granulosa cells (47). Treatment with TGF-β1 activates the Smad2/3 and ERK1/2 signaling pathways. Based on siRNA-mediated knockdown approaches and pharmacological inhibitors, those authors showed that activation of the Smad3 and ERK1/2 signaling pathways are needed for the TGF-β1–induced downregulation of StAR expression and a decrease in progesterone production. These results provide important insights into the molecular mechanisms that mediate TGF-β1–regulated StAR expression and progesterone production (47). Park et al. (48) found that the inhibitory effect of TGF-β1 on testicular steroidogenesis occurs through the cross-talk of TGF-b1/ALK5-activated Smad3 with orphan nuclear receptor Nur77. Consequently, Smad3 indirectly regulates the promoter activity of steroidogenic genes. These findings provide a molecular mechanism for TGF-β1-mediated repression of testosterone production in testicular Leydig cells (48).

Adrenocortical cells possess TGFβl receptors (mainly type I: TGFBR1 and type III: TGFBR3), which increase in number in response to ACTH treatment. Binding of mature TGFβl to its adrenocortical cell receptors causes numerous intracellular biochemical events resulting in inhibited cortisol production. Two important targets negatively regulated by TGFβl are angiotensin II receptors and the steroid 17a-hydroxylase (cytochrome P-45017) enzyme (49).

Currently, it is accepted that adropin exerts its biological role through activation of the GPR19 receptor, which belongs to the G-protein-coupled receptor family (GPCR) (50). The current model of GPCR signaling includes three main pathways: (1) the classical pathway, where appropriate ligand binding leads to downstream activation of G protein and other effector molecules, (2) arrestin signaling via ligand-regulated scaffolds (51), and (3) transactivation of protein serine/threonine kinase receptors (PS/TKRs), including TGFBR1, through the receptors of GPCR agonists, a mechanism first described by Ullrich in 1996 (52, 53). Our results suggest that adropin exerts its inhibitory effect on HAC15 cell steroidogenesis by binding to the GPR19 receptor and then stimulating the TGF-β-dependent pathway via transactivation. Based on experiments using the TGFβl kinase inhibitor, we confirmed that attenuation of steroidogenesis caused by adropin is mediated by the TGF-β signaling pathway, confirming the adropin-dependent transactivation mechanism. Other GPCR agonists such as thrombin (54), factor X (55), LPA (56) and endothelin-1 (57) lead to time-dependent activation of the TGF-β dependent signaling pathway. Previous research has shown that in vascular smooth muscle cells thrombin transactivation of the TGFBR1 based on cytoskeletal reorganization that activates Ras homolog member A (RhoA)/Rho-associated, coiled-coil containing protein kinase (ROCK) signaling, triggering the activation of integrin-dependent signaling and finally activating the large latent complex which holds TGF-β near the cell surface with the potential for activation of TGFBR1 (54, 58). Although our microarray study showed that adropin did not affect the expression of the RhoA/ROCK genes in the HAC15 cells, their expression was relatively high (data not shown), indicating that this intracellular mechanism of TGFBR1 transactivation may also occur in the HAC15 cell line. However, this hypothesis requires further study for confirmation.

We used the GSEA approach to identify numerous genes with increased expression due to activation of the TGF-β dependent pathway; however, we also identified genes that lead to increased TGF-β signaling pathway activity. Previous research has shown that silencing the dachshund family transcription factor 1 (DACH1) in H295R cells increases aldosterone production by suspending the TGF-β signaling pathway, whereas overexpression of DACH1 caused decreasing in aldosterone biosynthesis by activating the TGF-β dependent pathway (59). In the context of our study, incubation of HAC15 cells with adropin significantly stimulated DACH1 gene expression (fold = 1.64, adj. *p* = 0.04), which in turn may boost the inhibitory effect of TGF-β on HAC15 cell steroidogenesis.

The available published data indicate that adropin exerts a stimulating effect on the proliferative activity of a variety of cells. Among other mechanisms, it has been shown that adropin stimulates 3T3-L1 (fibroblasts) and rat primary preadipocyte cell proliferation via the ERK1/2 and AKT signaling pathways. (34). Adropin stimulate proliferation in mesenchymal-like MDA-MB-231 cells by MAPK/ERK1/2 signaling pathway which is essential for the upregulation of E-cadherin and accompanying phenotypic changes in human breast cancer cells (11). Moreover, adropin upregulates—both *in vitro* and *in vivo*—the expression of eNOS in endothelial cells. Furthermore, endothelial cells exposed to adropin show increased proliferation and migration, higher capillary-like tube formation rates, decreased permeability, and less TNF-alpha induced apoptosis (8). By contrast, Sato et al. demonstrated that in human aortic smooth muscle cells (HASMCs), adropin suppressed cell migration and proliferation without inducing apoptosis *via* ERK1/2 and Bax downregulation (60). It is important to emphasize that our results showed that exposing HAC15 cells to adropin stimulates the proliferative activity of HAC15 cells via ERK1/2 and AKT mediated pathway (Figure 11B). Moreover, the stimulatory effect of adropin on the proliferation of these cells was long-lasting, persisting throughout the entire time (50 h after administration) the cells were cultured in the presence of the peptide.

Because of stimulatory effect of the adropin on proliferation of human derived adrenocortical carcinoma cell line occurred via ERK1/2 and AKT dependent mechanism, we decided to analyse the clinical significance of the obtained results. According to the GEPIA data and our results, the expression of GPR19 was significantly elevated in adrenocortical carcinoma in relation to normal adrenal. Considering the stimulatory roles of adropin on HAC15 cell proliferation, we can assume that elevated expression of the GPR19 may indirectly cause a speed-up rate of proliferation in adrenocortical carcinoma. This suggestion seems to be confirmed in Kaplan–Meier survival analysis of 91 adrenocortical carcinoma samples from TCGA database. Increased expression of GPR19 leads to a statistically significant decrease in survival, suggesting that high expression of GPR19 could constitute a negative prognostic factor of adrenocortical carcinoma disease progression, however this aspect require further studies.

Most of the results discussed in this paper are based on experiments involving the administration (both *in vivo* and *in vitro*) of exogenous adropin. The physiological role of this peptide in the body remains an open question. Experiments on adropin knockout mice (AdrKO) provide some information in this respect, with published data showing that adropin prevents insulin resistance, dyslipidemia, impaired glucose tolerance, and loss of T lymphocytes (3, 61). The role of GPR19, the putative adropin receptor, is also debatable and not fully understood. In this respect, Martin (62) performed a study to evaluate agonist-independent constitutive signaling of several GPCR receptors. Using this method, the GPR19 receptor was classified as a “non-responder,” with “revealed patterns that suggest either a lack of constitutive signaling or an unresolved triggering condition (i.e., agonist dependent)” (62). The identification of a specific adropin antagonist would possible provide a better understanding of the role of this peptide in cells, organs, and in the entire body.

## Conclusions

In this study, we investigated the influence of exogenous adropin on proliferation and steroidogenesis in the human HAC15 adrenal carcinoma cell line. We found that exposing HAC15 cells to adropin decrease the secretion of aldosterone and cortisol by HAC15 cells. This effect was accompanied by inhibition of both StAR and CYP11A1 expression. Bioinformatic analysis based on microarray data suggests that adropin-mediated inhibition of adrenocortical hormone biosynthesis involves activation of the TGF-β signaling pathway likely to act through transactivation mechanism. We also found that HAC15 cells treated with adropin presented significantly higher proliferation rate than untreated cells. Using specific intracellular inhibitors, we showed that adropin stimulated proliferation occurs via ERK1/2 and AKT dependent signaling pathways. GPR19 expression was elevated in human adrenocortical carcinoma in relation to normal adrenals. High level of GPR19 could constitute a negative prognostic factor of disease progression.

## Data Availability Statement

The datasets presented in this study can be found in online repositories. The names of the repository/repositories and accession number(s) can be found in the article/Supplementary Material.

## Ethics Statement

The studies involving human participants were reviewed and approved by Bioethics Committee of Poznan University of Medical Sciences. The patients/participants provided their written informed consent to participate in this study.

## Author Contributions

ES, PM, and HK invented and designed the experiments, performed the experiments, analyzed the data, and prepared the manuscript. KJ performed the microarray experiments. MT and MS performed the experiments and performed QPCR. ML and WSu performed the experiments. BS and KB performed the experiments and analyzed the data. MRuch, MK, and TW designed the experiments and performed the experiments. WSz designed the experiments, performed the experiments, analyzed the data, prepared graphical presentation, and prepared the manuscript. LM invented and designed the experiments, analyzed the data, and prepared the manuscript. MRuci invented and designed the experiments, analyzed the data, prepared graphical presentation, and prepared the manuscript. All authors contributed to the article and approved the submitted version.

## Conflict of Interest

The authors declare that the research was conducted in the absence of any commercial or financial relationships that could be construed as a potential conflict of interest.

## Supplementary Material

Supplementary Figure 1 Expression of ARRDC3, JUN, HIST1H2BM and KIF20A genes in the HAC15 cell line after 24 h of incubation with ACTH, forskolin and adropin versus control (untreated) cells. Real time QPCR study (*N* = 4/group). Statistical differences were determined by the Mann–Whitney U non-parametric test compared to controls. ^*^*p* < 0.05; ^**^*p* < 0.01.
