# Supplementary figures and images for: Adropin Stimulates Proliferation and Inhibits Adrenocortical Steroidogenesis in the Human Adrenal Carcinoma (HAC15) Cell Line
Source: Front Endocrinol (Lausanne). 2020 Oct 8;11:561370. doi: 10.3389/fendo.2020.561370 (PMC7579427; doi:10.3389/fendo.2020.561370)

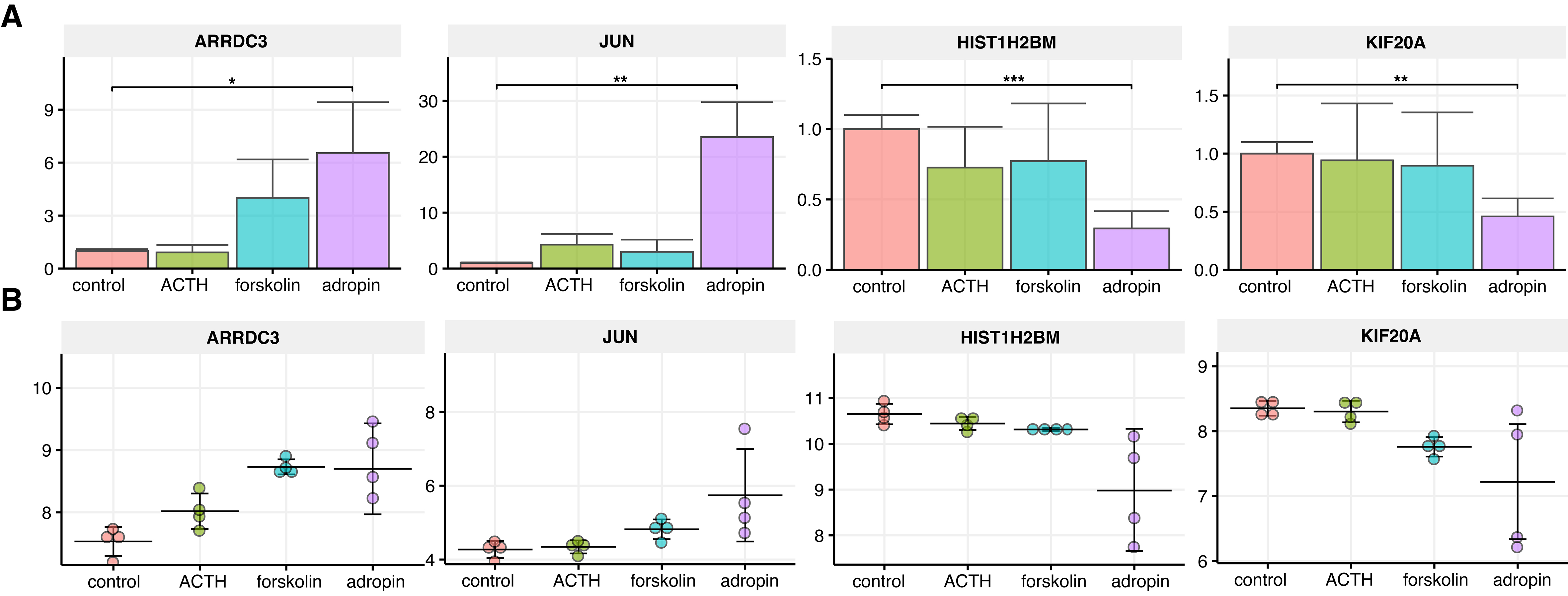

Supplement: Supplementary Figure 1 — Expression of ARRDC3, JUN, HIST1H2BM and KIF20A genes in the HAC15 cell line after 24 h of incubation with ACTH, forskolin and adropin versus control (untreated) cells. Real time QPCR study (N = 4/group). Statistical differences were determined by the Mann–Whitney U non-parametric test compared to controls. *p < 0.05; **p < 0.01. [file Image_1.JPEG]
